# Supplementary material for: Tailoring the Emission Wavelength of Color Centers in Hexagonal Boron Nitride for Quantum Applications
Source: Nanomaterials (Basel). 2022 Jul 15;12(14):2427. doi: 10.3390/nano12142427 (PMC9323195; doi:10.3390/nano12142427)
Supplement: Supplementary file 1 [file nanomaterials-12-02427-s001.zip › nanomaterials-1804730-supplementary.pdf]

Supplementary Information: Tailoring the emission wavelength of color centers in hexagonal boron nitride for quantum applications

Chanaprom Cholsuk, Sujin Suwanna, and Tobias Vogl

1 Summary of all studied defects

Supplementary File S1 summarizes all studied defects (sorted by type and by wavelength) in terms of electronic transition type, transition energy, wavelength, and lattice deformation.
It is accessible via https://doi.org/10.5281/zenodo.6826694.

2 Simplified electronic structures in each periodic group

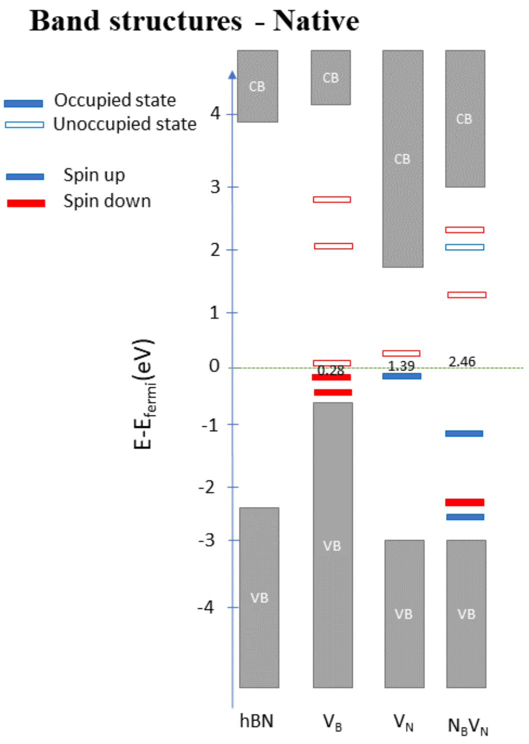

Figure S1: Simplified electronic structures for native defects.

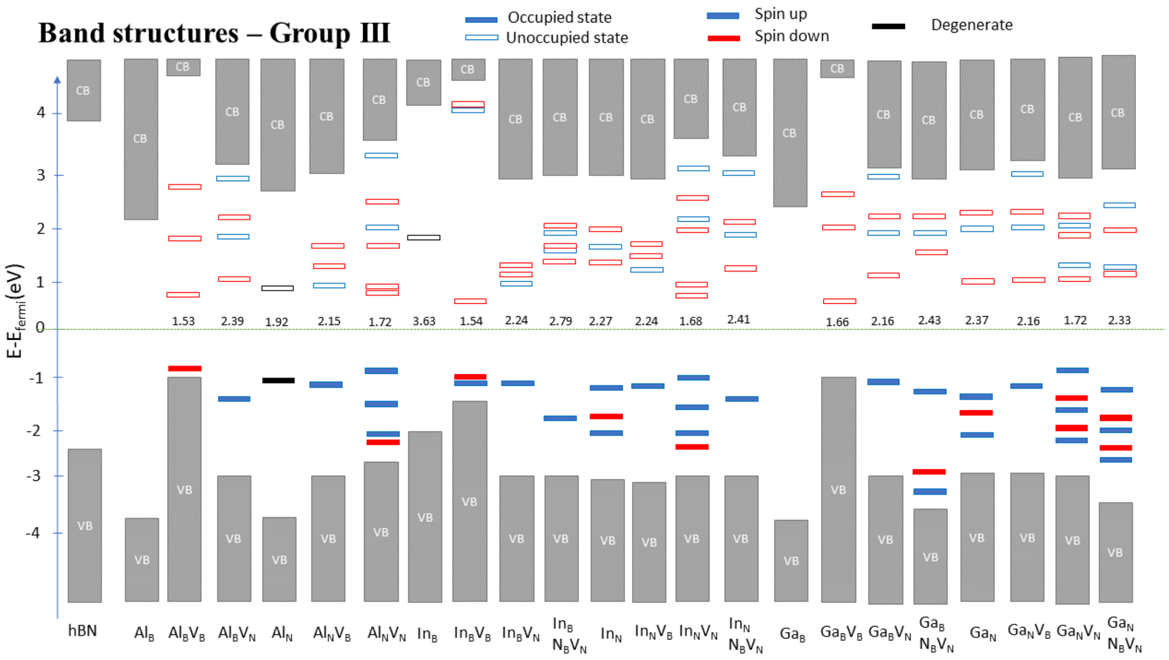

Figure S2: Simplified electronic structures for periodic group III.

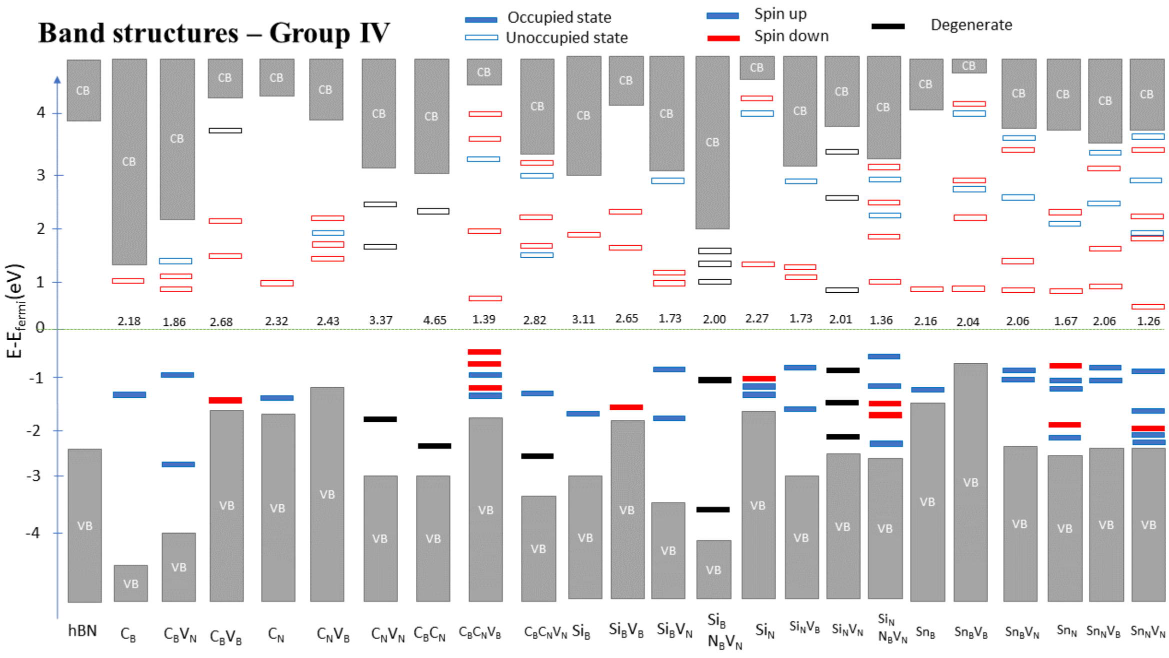

Figure S3: Simplified electronic structures for periodic group IV.

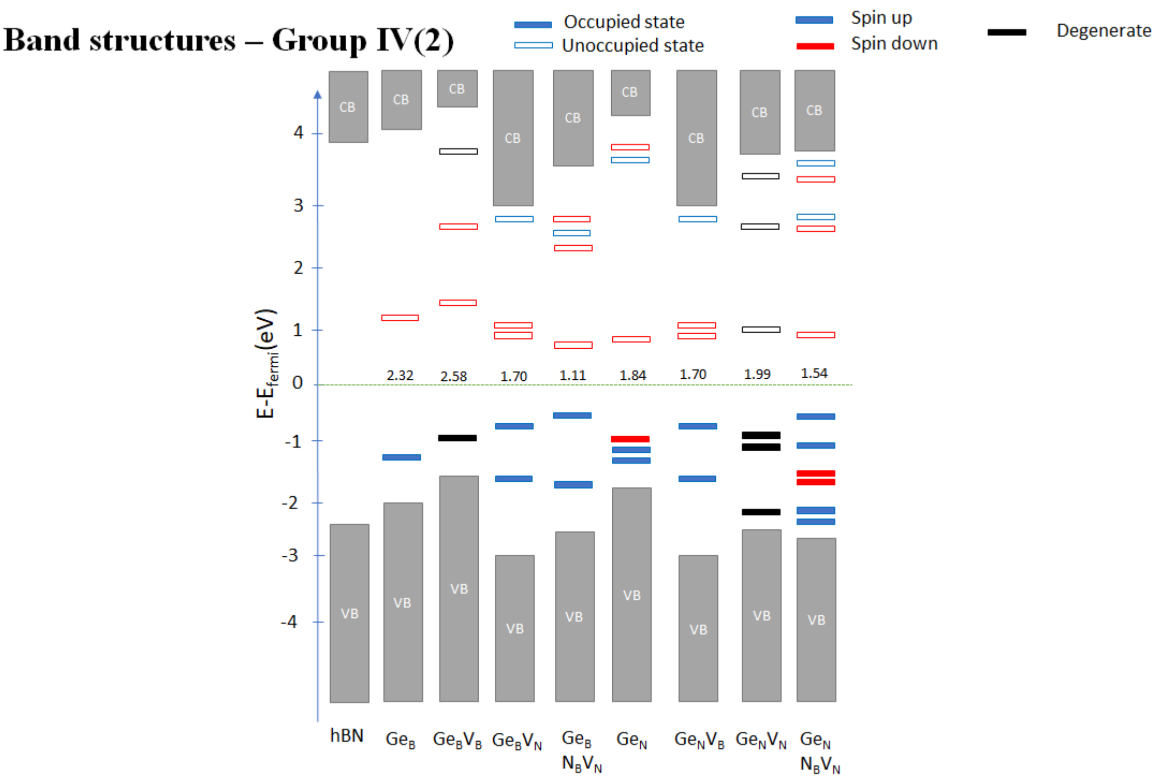

Figure S4: Simplified electronic structures for periodic group IV.

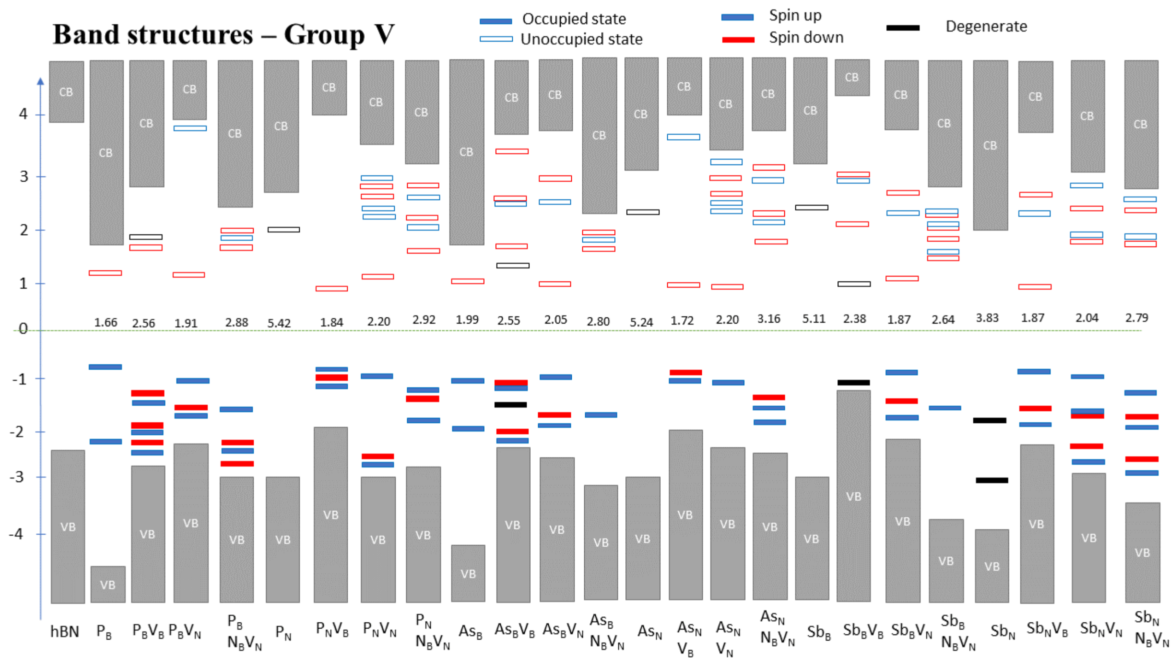

Figure S5: Simplified electronic structures for periodic group V.

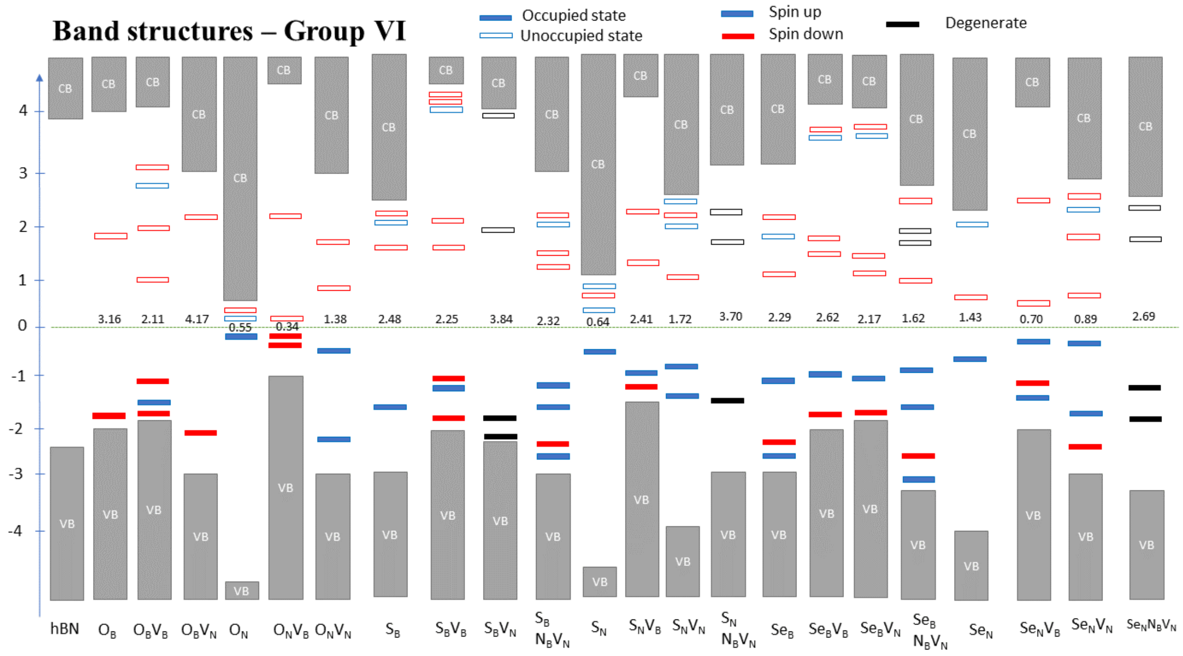

Figure S6: Simplified electronic structures for periodic group VI.

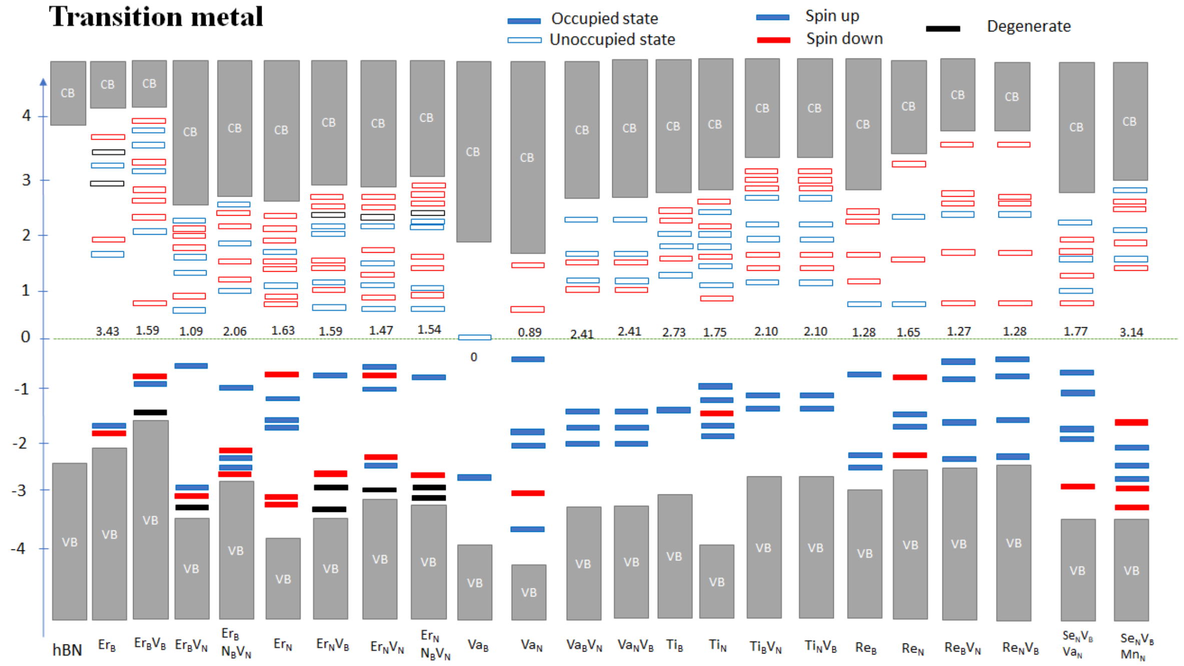

Figure S7: Simplified electronic structures for transition metals (TM).

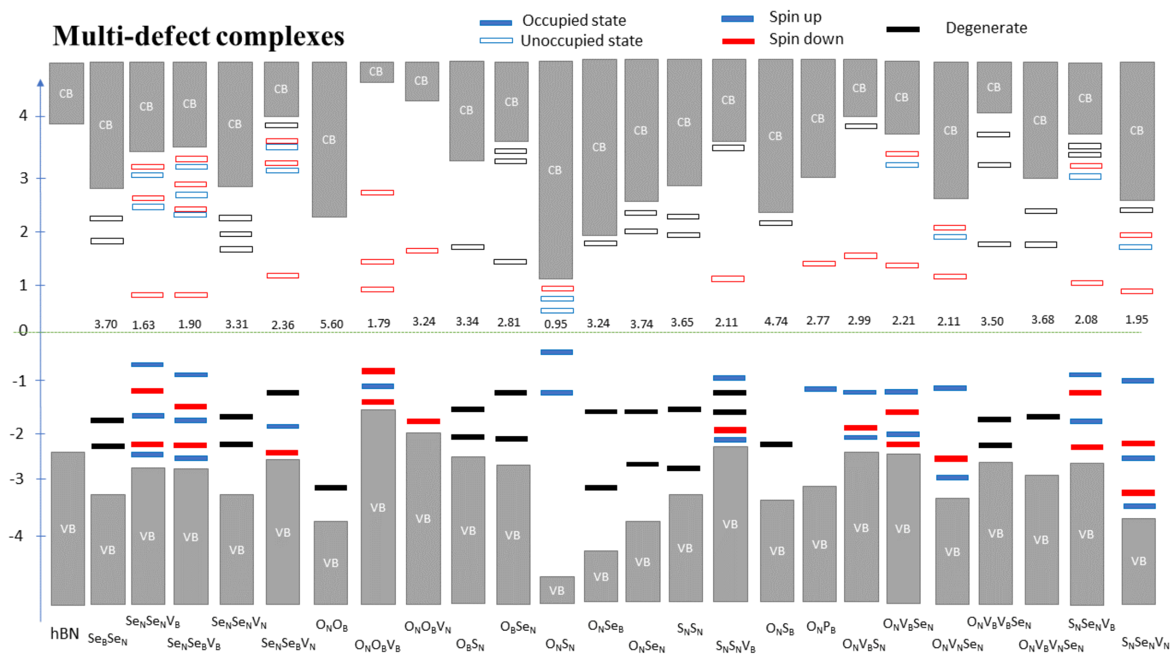

Figure S8: Simplified electronic structures for multi-defect complexes.

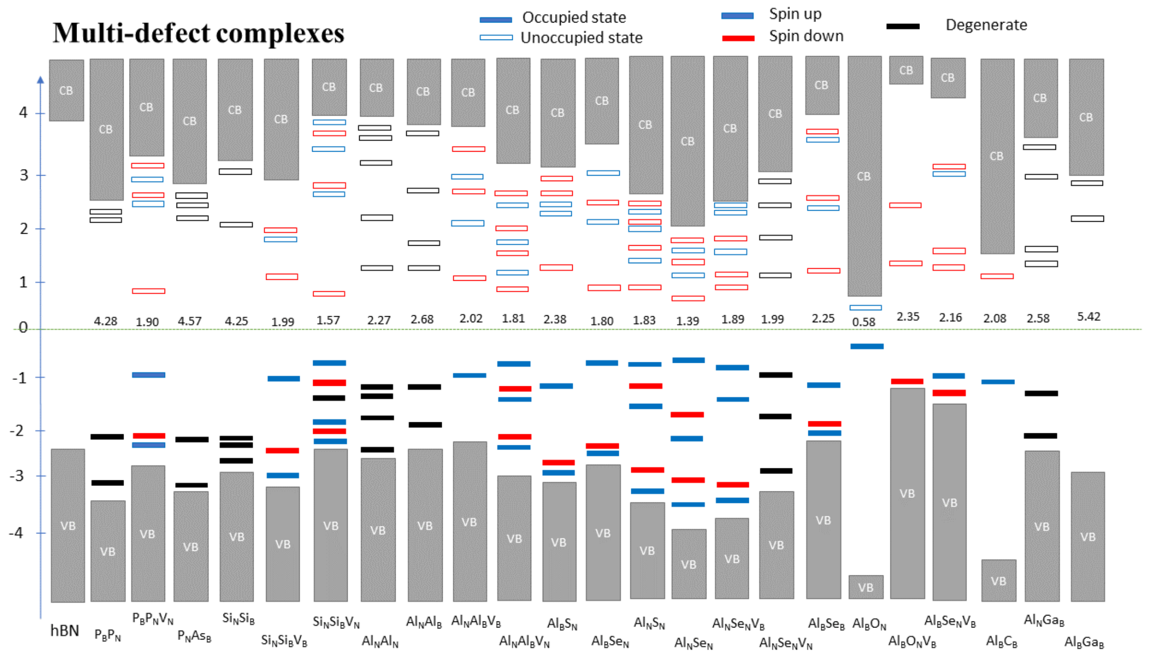

Figure S9: Simplified electronic structures for multi-defect complexes.

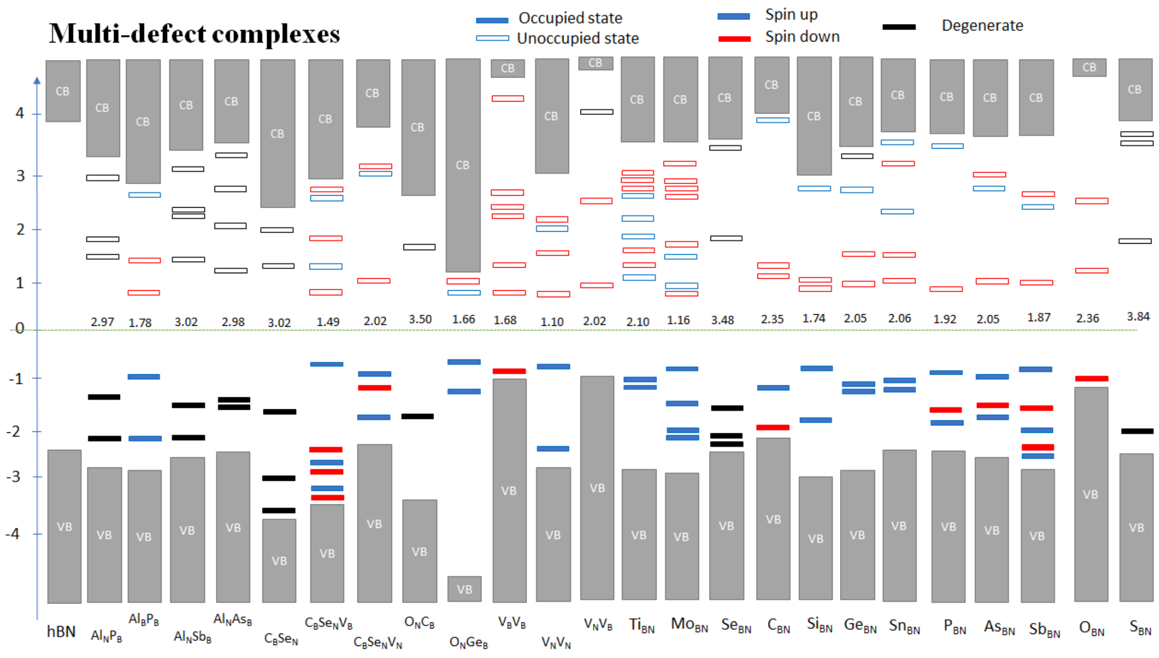

Figure S10: Simplified electronic structures for multi-defect complexes.

3 Effect of strain on electronic band structures

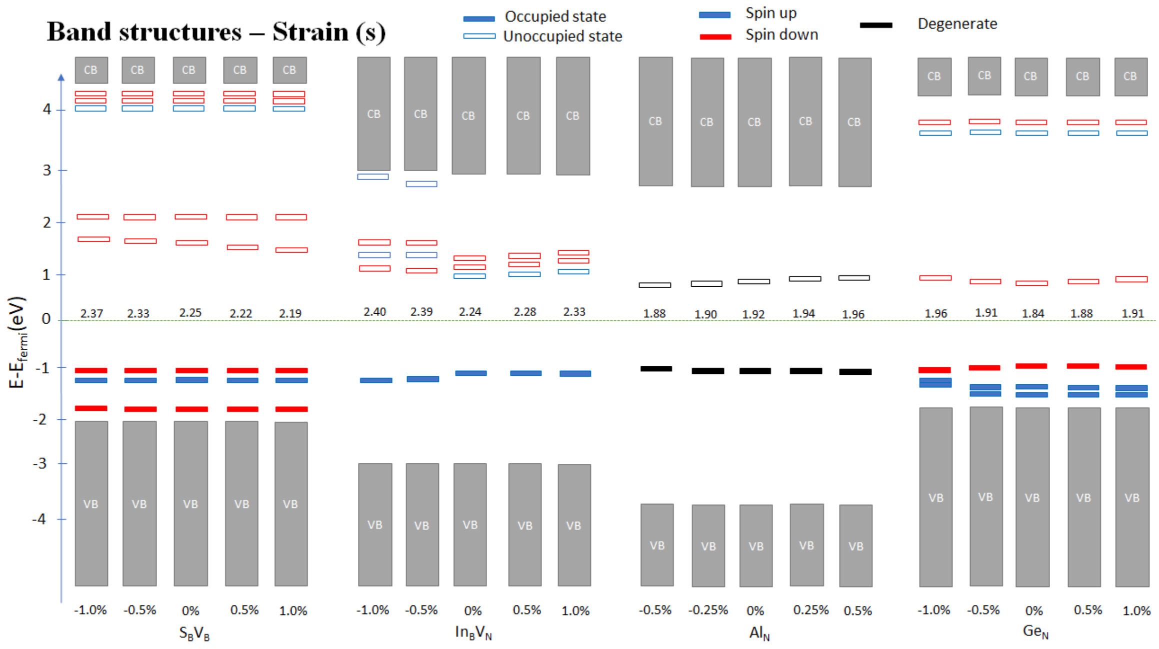

Figure S11: Simplified electronic structures of defects affected by bi-axial strain.

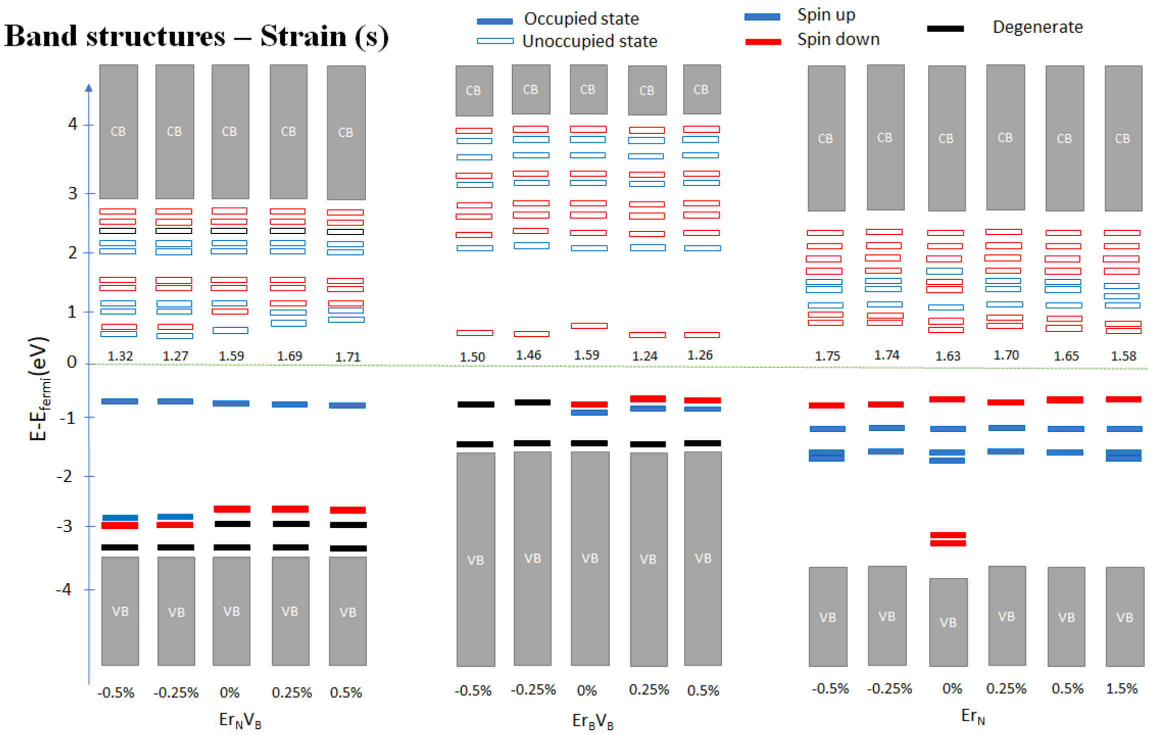

Figure S12: Simplified electronic structures of defects affected by bi-axial strain.

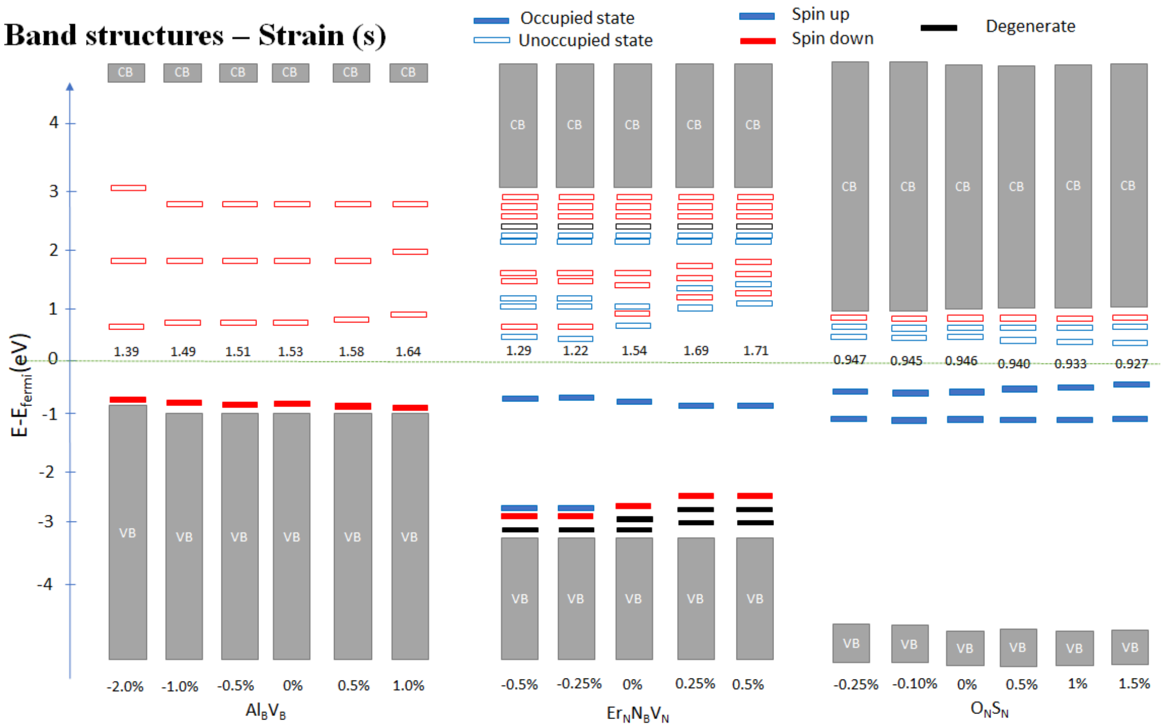

Figure S13: Simplified electronic structures of defects affected by bi-axial strain.

4 Effect of charge on electronic band structures

Table S1: Charged-state defects with transition types. ET , λ , and ↓ - ↑ denote the transition energy, wavelength, and the spin-flipping transition, respectively. Transitions from valence band (VB), or to conduction band (CB), or between generate states (deg) are also listed. Radiative transitions are shaded in gray.

Defect	Charge -1			Neutral charge			Charge +1		
	E_T (eV)	λ (nm)	Transition	E_T (eV)	λ (nm)	Transition	E_T (eV)	λ (nm)	Transition
Er_N	1.361	911.1	↓ - ↓	1.634	758.7	↓ - ↓	1.607	771.6	↑ - ↓
Er_BV_B	1.030	1204.3	↓ - ↓	1.592	778.7	↓ - ↓	1.398	886.9	deg - ↓
Si_N	3.852	321.9	deg - CB	2.268	546.8	↓ - ↓	-	-	-
Ti_B	2.410	514.5	↑ - ↑	2.733	453.6	↑ - ↑	2.053	604.1	VB - deg
Re_B	1.331	931.5	↑ - ↑	1.277	971.4	↑ - ↑	2.869	432.3	↑ - ↓
Er_B	1.898	653.3	↑ - ↓	3.433	361.2	↑ - ↑	0.789	1572.3	VB - ↓
$Er_BN_BV_N$	1.205	1028.7	↑ - ↑	2.063	601.1	↑ - ↑	2.980	416.1	VB - ↑
Er_NV_B	0.734	1690.2	↑ - ↓	1.590	779.6	↑ - ↑	3.122	397.2	VB - ↑
Er_BV_N	1.427	869.0	↓ - ↓	1.089	1138.7	↑ - ↑	3.124	396.9	VB - ↑
C_NV_B	2.100	590.4	↑ - ↓	2.433	509.8	VB - ↓	2.042	607.1	↓ - deg
As_N	1.131	1096.4	↑ - ↓	5.24	236.6	deg - deg	4.813	257.6	↓ - deg
Si_BV_N	1.796	690.4	↑ - ↓	1.734	715.2	↑ - ↓	2.058	602.4	↑ - ↑
$Si_NN_BV_N$	2.070	599.2	↑ - deg	1.360	912.0	↑ - ↓	1.808	685.8	↑ - ↓
Sn_BV_N	2.198	564.1	↑ - ↓	2.057	602.7	↑ - ↓	1.760	704.5	↑ - ↓
Al_NV_N	1.715	723.2	↑ - ↓	1.72	721.3	↑ - ↓	2.248	551.6	↑ - ↓
In_N	-	-	-	2.27	547.3	↑ - ↓	-	-	-
Ga_BV_N	2.870	432.1	deg - CB	2.16	574.3	↑ - ↓	3.395	365.2	VB - deg
P_B	0.312	3976.0	↓ - CB	1.66	748.8	↑ - ↓	2.272	545.9	VB - ↓
Sb_NV_B	2.977	416.6	deg - CB	1.87	661.5	↑ - ↓	0.829	1495.3	↑ - ↓
O_NV_N	1.882	659.0	↑ - ↓	1.38	896.6	↑ - ↓	2.105	588.9	↑ - ↓
Se_NV_N	1.868	663.8	↑ - ↓	0.89	1392.4	↑ - ↓	2.202	563.1	↑ - ↓
S_NV_N	1.823	680.3	↑ - ↓	1.72	720.1	↑ - ↓	2.182	568.2	↑ - ↓
Re_BV_N	2.261	548.4	↑ - ↓	1.265	979.7	↑ - ↓	1.158	1070.4	↑ - ↓
Ti_N	1.629	761.3	↑ - ↓	1.7535	707.2	↑ - ↓	2.014	615.7	↑ - ↓
$Al_NSe_NV_B$	1.719	721.5	↑ - ↓	1.886	657.5	↑ - ↓	2.284	542.9	↑ - ↓
C_B	0.814	1523.0	deg - CB	2.18	568.7	↑ - ↓	4.112	301.6	VB - deg

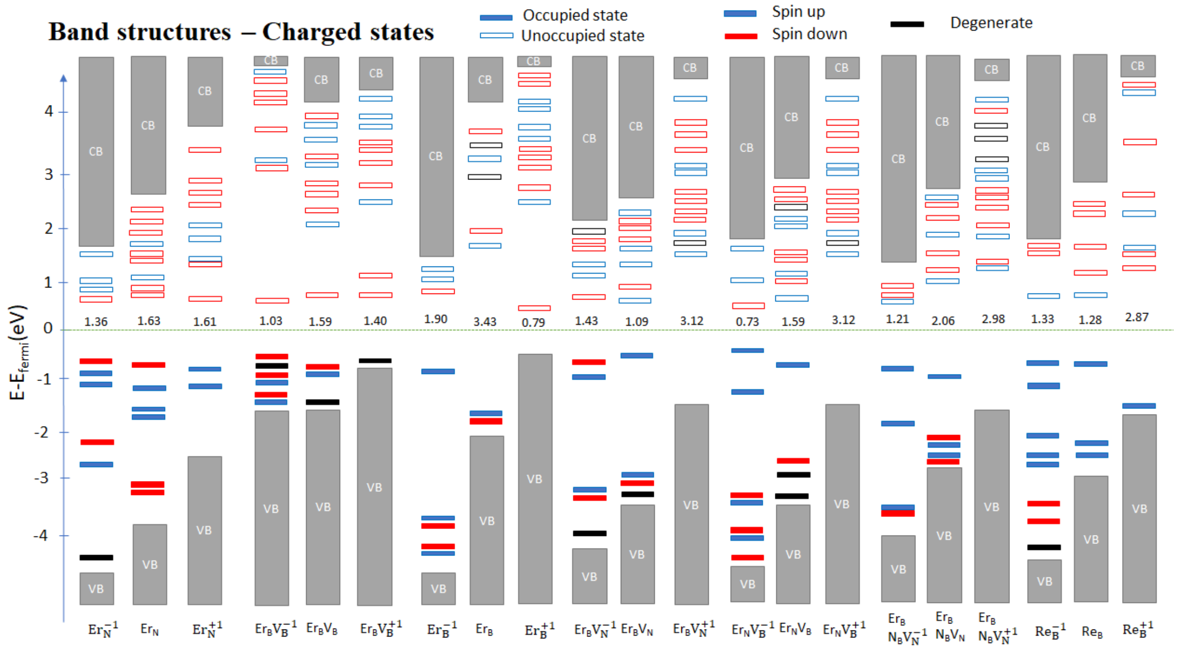

Figure S14: Simplified electronic structures for charged-state defects.

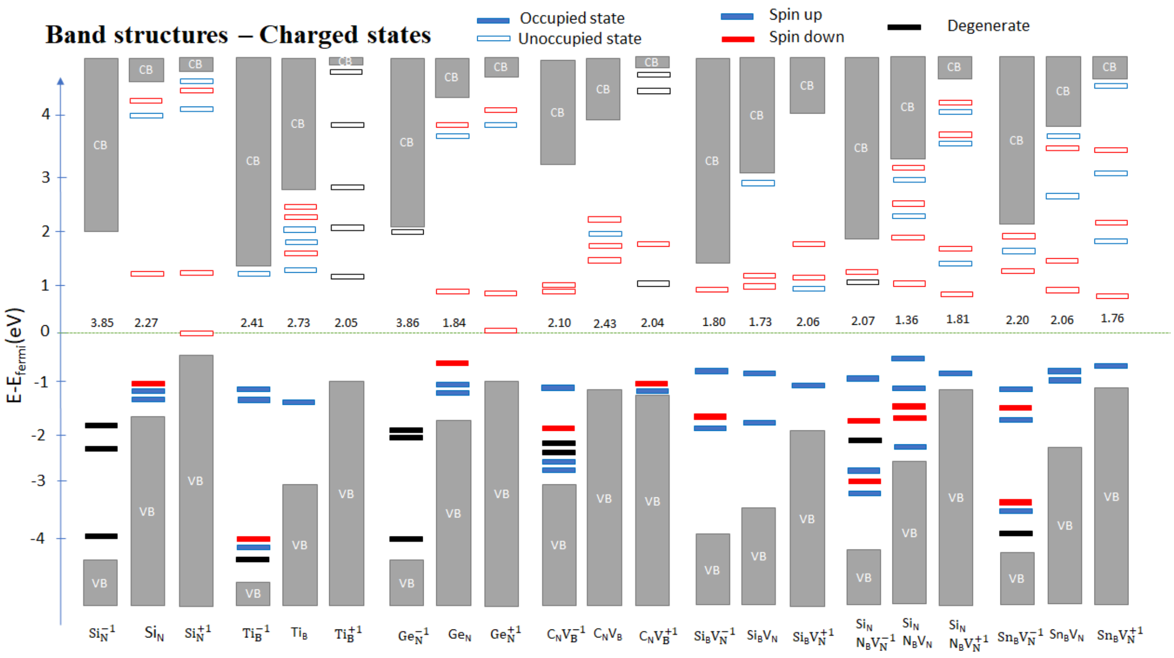

Figure S15: Simplified electronic structures for charged-state defects.

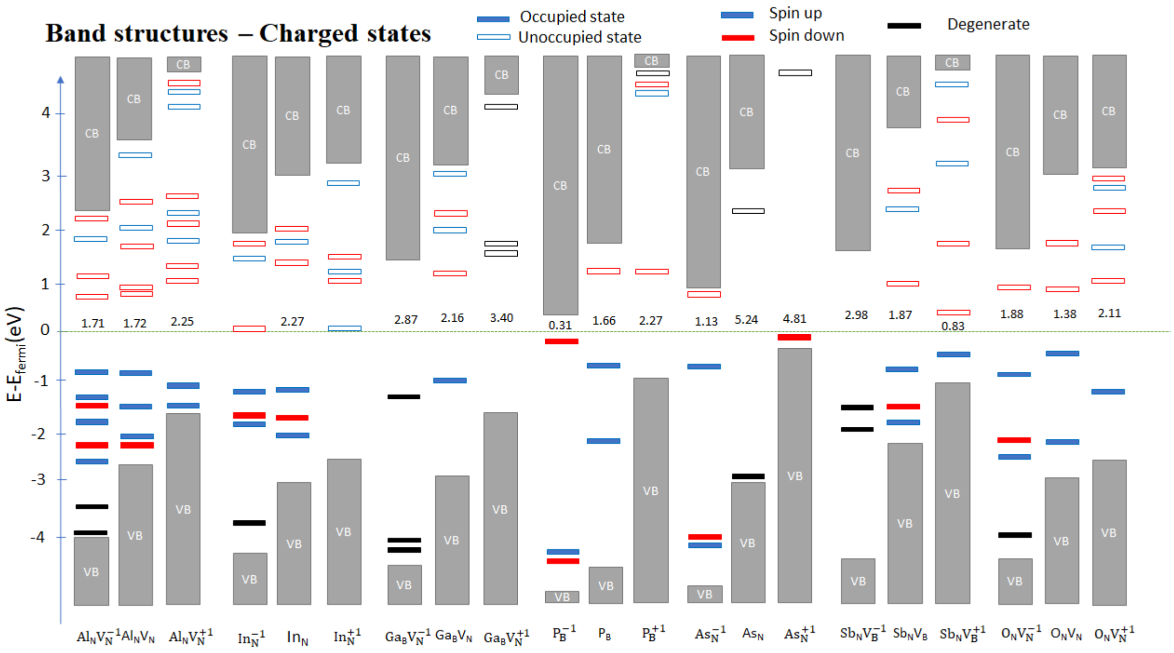

Figure S16: Simplified electronic structures for charged-state defects.

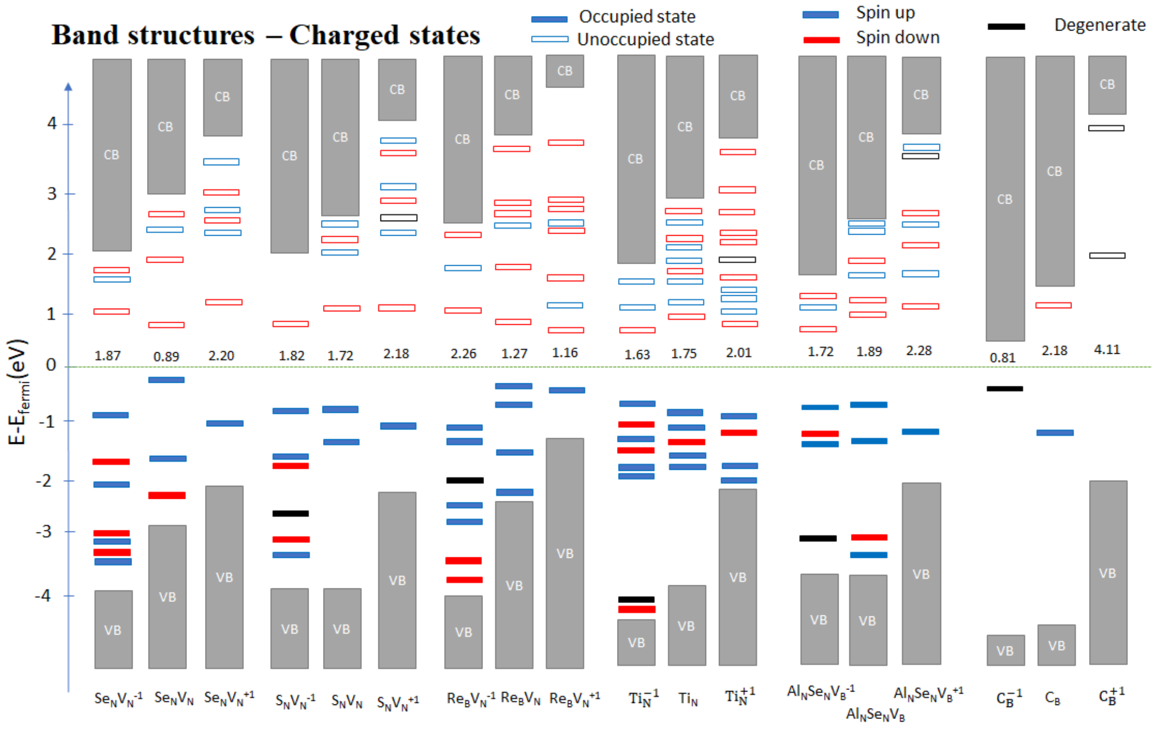

Figure S17: Simplified electronic structures for charged-state defects.
